# A comparison of single-dose and multiple divided daily-dose oral steroids for sudden sensorineural hearing loss^☆^

**DOI:** 10.1016/j.bjorl.2018.06.001

**Published:** 2018-07-17

**Authors:** Gun Hee Yu, Yong-Jun Choi, Hahn Jin Jung, Yun-Sung Lim, Seok-Won Park, Chang Gun Cho, Joo Hyun Park

**Affiliations:** a Ilsan Hospital, Dongguk University, College of Medicine, Department of Otorhinolaryngology-Head and Neck Surgery, Goyang, Korea; b Chungbuk National University Hospital, Chungbuk National University College of Medicine, Department of Otorhinolaryngology-Head and Neck Surgery, Cheongju, Korea

**Keywords:** Sudden sensorineural hearing loss, Steroid, Dose, Regimen, Perda auditiva neurossensorial súbita, Esteroide, Dose, Regime

## Abstract

**Introduction:**

Glucocorticoids are considered the first-line therapy for sudden sensorineural hearing loss. But there is currently no consensus on administering them as a single dose versus multiple divided daily doses.

**Objective:**

We aim to evaluate the treatment outcome of sudden sensorineural hearing loss between a single-dose and multiple divided daily doses of steroid treatment.

**Methods:**

A total of 94 patients who were diagnosed and treated for sudden sensorineural hearing loss and followed up for more than three months were reviewed retrospectively. Patients were divided into single-dose and multiple divided-dose groups, based on their medication regimens. Hearing thresholds were repeatedly measured: on the initial visit and 1 week, 1 month, and 3 months after the initial treatment. Treatment outcomes were analyzed by comparing hearing recovery rates and post-treatment audiometric changes.

**Results:**

The hearing threshold was significantly reduced at three months post-treatment in both groups. The hearing recovery rate of the single-dose group was significantly higher than that of the multiple divided-dose groups. Audiometric changes showed no statistical difference either in pure tone threshold or speech discrimination.

**Conclusion:**

When oral steroids are indicated for sudden sensorineural hearing loss, both a single dose and multiple divided doses can be effective for treatment and have comparable results. However, the single-dose regimen seems to be more efficacious than the divided-dose regimen.

## Resumo

**Introdução:**

Os glicocorticoides são considerados terapia de primeira linha para perda auditiva neurossensorial súbita. Contudo, atualmente não há consenso em como para administrá-los, se em dose única ou múltiplas doses diárias.

**Objetivo:**

Nosso objetivo é avaliar o resultado do tratamento da perda auditiva neurossensorial súbita com uma dose única ou várias doses diárias de tratamento com esteróides.

**Método:**

Um total de 94 pacientes que foram diagnosticados e tratados para perda auditiva neurossensorial súbita e acompanhados por mais de três meses pós-tratamento foram avaliados retrospectivamente. Os pacientes foram divididos em grupos de dose única diária e dose diária dividida em múltiplas tomadas, baseado em seu regime medicamentoso. Os limiares auditivos foram medidos repetidamente: na visita inicial e em 1 semana, 1 mês e 3 meses após o tratamento inicial. Os resultados do tratamento foram analisados comparando-se as taxas de recuperação da audição e as alterações audiométricas pós-tratamento.

**Resultados:**

O limiar auditivo foi significativamente reduzido aos três meses pós-tratamento em ambos os grupos. A taxa de recuperação auditiva no grupo de dose única foi significativamente maior do que no grupo de dose diária dividida em múltiplas tomadas. As alterações audiométricas não mostraram diferença estatística, tanto no limiar de tom puro quanto na discriminação da fala.

**Conclusão:**

Quando esteroides orais são indicados para perda auditiva neurossensorial súbita, tanto uma dose única quanto múltiplas doses podem ser eficazes para o tratamento e têm resultados comparáveis. No entanto, o regime de dose única diária parece ser mais eficaz do que o regime de dose diária dividida em múltiplas tomadas.

## Introduction

Glucocorticoids are considered a first-line therapy for sudden sensorineural hearing loss (SSNHL) and may be administered systemically (generally orally). Although the mechanism of the steroid action in the inner ear remains unclear, high concentrations of steroids in the inner ear via high doses of oral steroids have shown an adequate therapeutic effect.^1^ However, there is currently no consensus whether giving a single dose versus multiple divided daily doses of glucocorticoids is preferable.

The advantages of single-dose regimen are less suppression of hypothalamic–pituitary–adrenal function^2^ and high peak plasma concentration.^3^ Notably, the divided-dose regimen also has advantages, including high mean plasma concentration^3^ and a smaller number of tablets in each dose.

It is currently unclear as to which is more important—either peak or mean plasma concentration—regarding steroid treatment of SSNHL, and the efficacy of each regimen has not yet been compared.

The AAO-HNS guidelines recommend the following single-dose regimen: prednisolone 1 mg/kg/day for 7–14 days, then taper medication over a similar period.^4^ Although a single dose each morning has been known to cause less adrenal suppression, daily doses of oral steroids are commonly divided in clinics to encourage patient compliance by simplifying the pill-taking process. The purpose of this study was to compare the treatment outcomes of SSNHL patients between a single dose and multiple divided doses of a steroid treatment.

## Methods

One hundred twenty-nine SSNHL patients (age >18 years), who were treated with oral steroids and had more than 3 months’ follow-up duration between July 2013 and May 2016, were included in this study. SSNHL was diagnosed in patients who had experienced their first episode of sudden hearing impairment of more than 30 decibels (dB) across three contiguous frequencies. Patients who were treated with either a combined intratympanic steroid therapy or a different treatment regimen, patients with combined chronic otitis media, those who were finally diagnosed as having either Meniere's disease, acoustic neuroma, or Ramsay Hunt syndrome, and subjects who did not return for follow up, were excluded.

The participants’ medical charts were retrospectively reviewed. Seventy (54%) of the patients were men and 59 (46%) were women. The mean age was 55.5 years (range: 18–79 years). The right ear was affected in 55 cases, the left ear in 68 cases, and both ears in 7 cases. Patients were divided into two groups based on steroid regimens:

Single-dose group: prednisolone 1 mg/kg (or methylprednisolone 0.8 mg/kg) once a day for 5 days, and tapering doses (0.8 mg/kg, 0.6 mg/kg, 0.4 mg/kg, and 0.2 mg/kg for 2 days, sequentially).^5^

Multiple divided-dose group: the same dose in three equally divided doses, three times a day and tapering doses.

Pure tone audiometry (PTA) and speech audiometry were performed at the initial visit and 1 week, 2 weeks, 1 month, 2 months, and 3 months after the initial treatment. The PTA threshold was calculated by the mean hearing threshold frequencies (500, 1000 (×2), 2000 (×2), 4000 Hz). The speech discrimination (SD) score was obtained by speech audiometry (0–100%). These values were recorded at each follow-up visit, and the results of the initial and the last follow-up were evaluated. Hearing improvement was evaluated according to Siegel's criteria of hearing recovery in sudden deafness (Table 1).^6^ Complete, partial, and slight recovery were considered hearing recovery.

**Table 1:** Siegel's criteria.^6^

Siegel's criteria	
Complete recovery	Final hearing better than 25 dB
Partial recovery	More than 15 dB gain, final hearing 25–45 dB
Slight recovery	More than 15 dB gain, final hearing poor than 45 dB
No improvement	Less than 15 dB gain or final hearing poor than 75 dB

Comparisons between pre- and post-treatment PTA and SD in each group were performed using the paired *t*-test. The Chi-square test was used to compare the hearing recovery rate between the two groups, and the Student *t*-test was used to evaluate the difference between the pre- and post-treatment PTA threshold and SD between the two groups Statistical analyses were performed using the Statistical Package for the Social Sciences (SPSS) for Windows version 18.0. The criterion for statistical significance was set at *p* < 0.05.

This study was approved by the Institutional Review Board of Our Hospital (ID no. 2017-08-013).

## Results

The single-dose group included 34 patients, and 95 patients were assigned to the divided-dose group, according to their steroid regimen. Demographics of each group are summarized in Table 2. Pre-treatment PTA thresholds were 70.8 dB and 70.6 dB in the single-dose group and the divided-dose group, respectively (*p* = 0.997). The SD scores were also not different between the groups before treatment. There were no significant differences in age, sex, incidences of associated vertigo, hypertension and diabetes *mellitus*, and interval of onset of hearing loss and steroid treatment between the two groups (Table 2).

**Table 2:** Demographic characteristics of patients in the single-dose and divided-dose group.

	Single-dose group (*n* = 34)	Divided-dose group (*n* = 95)	*p*-Value
Age (year)	55.4 ± 4.4	55.5 ± 3.0	0.965
Sex (M/F)	18/16	52/43	1.000
Pre PTA (dB)	70.8 ± 11.0	70.6 ± 5.4	0.977
Pre SD (%)	28.7 ± 12.6	28.4 ± 6.2	0.967
Dizziness	9	20	0.422
Hypertension	11	30	0.934
Diabetes mellitus	9	31	0.528
Interval of HL-Initial Tx (day)	3.4 ± 0.8	5.1 ± 1.4	0.027

PTA, Pure Tone Audiometry; SD, Speech Discrimination; HL, Hearing Loss, Tx, Treatment.

Data is presented as mean ± 95% CI.

**Figure 1:**
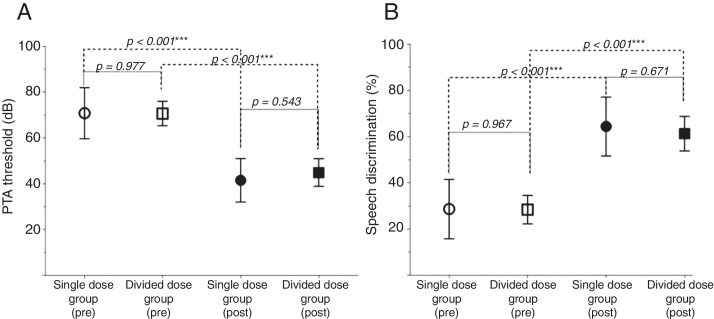
Pre- and post-treatment PTA threshold (A) and SD (B) in the two groups.

**Figure 2:**
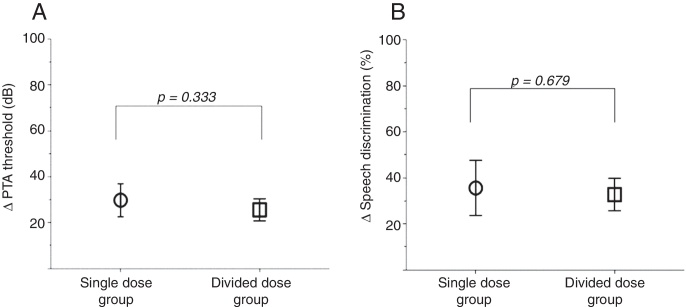
Comparison of change of the PTA threshold (A) and SD (B) between the two groups.

The hearing threshold and SD were significantly reduced at three months post-treatment in both groups (Fig. 1). However, post-treatment PTA and SD were not different statistically between the two groups (Fig. 2) (PTA, *p* = 0.543; SD, *p* = 0.671).

Thirty out of 34 patients in the single-dose group (complete recovery: 13, partial recovery: 4, slight recovery: 13), and 56 out of 95 subjects (complete recovery: 34, partial recovery: 8, slight recovery: 14) showed hearing improvement (Table 3). The hearing recovery rate of the single-dose group was significantly higher than that of the multiple divided-dose group (*p* = 0.003). When considering complete and partial recovery only, there was no significant difference in the hearing recovery rate between both groups (17/34 [50%] vs. 42/95 [59%], *p* = 0.317) (Table 3).

**Table 3:** The hearing recovery rate of the two groups.

	Single dose group (*n* = 34)	Divided dose group (*n* = 95)
Complete recovery	13 (38%)	34 (36%)
Partial recovery	4 (12%)	8 (8%)
Slight recovery	13 (38%)	14 (15%)
No improvement	4 (12%)	39 (41%)

Pre- and post-treatment changes of the PTA threshold and SD in each group were not different (*p* = 0.333 and 0.679, respectively). No severe side effect of the steroid was observed in any patient.

## Discussion

The etiology of SSNHL is still unclear. Although there are various hypotheses to explain the causes of SSNHL, including autoimmune reaction, metabolic problems, and trauma, it is known that viral and vascular factors, which cause insufficient blood supply to the cochlear, play central roles.^7^ Therefore, various therapies have been introduced to improve cochlear perfusion, with steroids proven as the most efficient.^8^ However, there is no established dosage regimen for oral steroids and no consensus on which is more important between peak and mean plasma concentrations of steroids in the treatment of SSNHL. Although the AAO-HNS guidelines recommend a single-dose regimen,^4^ physicians still often prescribe a divided-dose regimen due to various concerns, such as complications with single high-dose steroids and patient compliance. Additionally, clinicians who mainly encounter patients with SSNHL tend to be less attuned to medication-induced adrenal suppression.

There is little data in the literature about the comparison of efficacy between different oral steroid dose regimens on SSNHL. We believe that this is the first description of the favorable efficacy of a single-dose regimen compared to a divided-dose regimen of steroid therapy. In our study, we divided patients into two groups according to steroid regimen (single dose versus multiple divided doses). The therapeutic effects of regimens on SSNHL were evaluated via both pre- and post-treatment pure tone threshold and speech discrimination scores, according to Siegel's criteria.^6^ We found that the single-dose regimen had the advantage in hearing recovery when slight, partial, and complete recovery were defined as “hearing recovery” (88% vs. 29%). When slight recovery was excluded from “hearing recovery”, there was no statistical difference between the two groups (50% vs. 42%). Therefore, we should be more cautious when asserting that hearing recovery is significantly better with a single-dose regimen than with a divided-dose regimen. SSNHL patients with profound hearing loss in the single-dose group showed a mean pre- and post-treatment PTA threshold of 105.6 and 70.9, respectively. Profound SSNHL patients in the divided-dose group showed pre- and post-treatment PTA threshold of 110.9 and 84.1, respectively. Only 2 of 27 patients with initial hearing threshold >90 dB showed serviceable hearing (PTA threshold <40 dB) after steroid treatment (complete recovery: 1, partial recovery: 1). When considering the improvement of hearing threshold (the change in PTA threshold), there was no significant difference between two groups (34.9 ± 6.9 vs. 27 ± 5.3, *p* = 0.338) in SSNHL patients with profound hearing loss.

The half-life of oral prednisolone was reported as 2.6–3.8 h, which varied by dose.^9^ At 60 mg, it was reported as 3.0 ± 0.4 h. Notably, peak concentration increases linearly but not directly proportional to dose size.^10^ With 60 mg of oral prednisolone, the peak concentration was reported as 520.0 ± 69.7 ng/mL, whereas total clearance was 10.9 ± 3.5 L/h.^11^ Therefore, it can be estimated that the single dose of the steroid had the advantage of a high peak plasma concentration, which was nearly eliminated before the next administration, while the same dose in three equally divided administrations had the advantage of a high mean plasma concentration. However, few studies have compared the effect of different dose regimens.

Recently, the comparison of the efficacy of two and three divided steroid regimens in SSNHL patients was reported.^12^ The authors demonstrated that the two divided dose regimen (40 mg of prednisolone in the morning, 20 mg at night) showed better outcomes in hearing recovery compared to the three divided dose regimen (20 mg of prednisolone three times daily).^12^ Regarding peak and mean concentration, our findings showed similar results to those of the previous study, and it is assumed that the peak plasma concentration of steroids may play an important role in the recovery of SSNHL. For a more reasonable comparison of peak and mean concentration, a single-dose regimen should be included, as was done in this study, because a single dose has a higher peak plasma concentration than a two divided dose regimen.

Forty patients with diabetes mellitus showed poor prognosis compared to patients without diabetes (*n* = 89, *p* = 0.003) and this result is consistent with previous studies.^13^ However, there was no statistically significant relationship between age (<60 or ≥60) and treatment outcome (*p* = 0.216) in our study. When comparing the recovery rate between SSNHL patients with vertigo (*n* = 30) and without vertigo (*n* = 99), the recovery rate was not statistically different (42% vs. 40%; *p* = 0.667).

Our study has some limitations. First, several suspected risk factors for SSNHL were not available from the retrospective data, including personal histories of smoking, exposure to noise, and cardiovascular risk factors, such as body mass index, cholesterol level, and a family history of cardiovascular disease. The data also lacked information about the participants’ immune statuses, which might have caused some bias. However, we confirmed that major risk factors, including age, hearing threshold at initial presentation, interval of hearing loss and treatment, and incidences of associated vertigo, hypertension and diabetes, were not different between the two groups before steroid therapy. And the majority of the included patients in this study have a PTA range of moderate-to-severe hearing loss. Second, a relatively small sample of single-dose group participants were enrolled compared to the divided-dose group. Because we tried to include patients in the same treatment period, and a substantial portion of patients who were treated with a single morning dose of steroids had intratympanic steroid therapy simultaneously, they were excluded from analysis. Third, SSNHL patients who did not receive steroid treatment could not be included. There was no statistically significant difference between the values of post-treatment hearing threshold of the two groups; therefore, the result can be interpreted differently if other criteria of hearing recovery are used.

The included patients had no serious side effect from the steroids; however, there were minor side effects, such as temporary elevation of blood pressure or blood sugar levels and gastrointestinal symptoms, in a few patients.

This study suggests the advantage of a single-dose regimen compared to multiple divided doses in patients with SSNHL. It is meaningful in that few studies have researched this topic previously. Further studies are needed to evaluate whether either peak or mean concentration of a plasma steroid is effective for a better outcome of hearing in SSNHL, including the long-term side effects of steroids, when considering these limitations

## Conclusion

When oral steroids are indicated for SSNHL, both a single dose and multiple divided doses can be effective and have comparable results. However, the single-dose regimen seems to be more efficacious than the divided-dose regimen in hearing recovery, even when considering the possibility of suppression of hypothalamic–pituitary–adrenal function.

## Conflicts of interest

The authors declare no conflicts of interest.
